# Tree holes as a source of water for primate species in an Amazonian Forest fragment, northern Brazil

**DOI:** 10.5194/pb-11-31-2024

**Published:** 2024-10-14

**Authors:** Luan Gabriel Araujo Goebel, Gabriela Rodrigues Longo, Marcela Alvares Oliveira, Manoel dos Santos-Filho, Raone Beltrão-Mendes

**Affiliations:** 1 Programa de Pós-Graduação em Ciências Ambientais, Universidade do Estado de Mato Grosso – UNEMAT, Cáceres, 78217-900, Mato Grosso, Brazil; 2 Instituto de Conservação de Animais Silvestres, Campo Grande, Mato Grosso do Sul, Brazil; 3 Programa de Pós-Graduação em Conservação e Uso de Recursos Naturais, Universidade Federal de Rondônia, Porto Velho, Rondônia, Brazil; 4 Programa de Pós-Graduação em Ecologia e Conservação, Universidade Federal de Sergipe, São Cristóvão, Sergipe, Brazil

## Abstract

Water is an essential nutrient for living beings and is fundamental to metabolic processes. Under free-living conditions, primate individuals can use different strategies, skills, and resources to access water. Here, we report on observations of water consumption in *Ateles chamek* and *Sapajus apella*; describe the environmental conditions in which such events were observed, as well as the behavior of the individuals; and compare these observations with similar records in neotropical primates. Water consumption was observed during primate surveys in a forest fragment of approximately 52 ha bordered by the Jaru River, located southwest of the Brazilian Amazon, Vale do Paraíso municipality, state of Rondônia, Brazil. To access water, individuals of *A. chamek* used their tails, whereas *S. apella* used a leaf as a tool. Our observations suggest that tree holes may be important water sources for primates in forest fragments and that individuals of different species use different strategies to collect water from tree holes. Access and consumption strategies are directly associated with different cognitive skills and behaviors, which may include using tools, as in the case of capuchins. As water consumption records are limited, these findings highlight the need for continuous reporting to better understand water acquisition. Such reports are especially needed in the context of fragmented and degraded habitats, where water availability is affected by edge effects and the reduction in both fleshy fruits and moisture, which are important for primate species.

## 1 Introduction

Water is an essential nutrient for life, given that it is mandatory for metabolic processes and the survival of species in the most varied environments (Jéquier and Constant, 2010). Many animal species obtain water directly from environmental sources, such as rivers, lakes, and puddles (Chaves et al., 2021). Other species obtain water through food intake (see Albuquerque et al., 2021). Primates, for example, commonly obtain the water necessary for their metabolic activities from food sources, mainly from fleshy fruits and young leaves (Chapman, 1988; Chaves et al., 2021). They can also obtain water from alternative sources, such as moisture on the surface of leaves (Milton, 1979; Nagy and Montgomery, 1980), sucking their hairs when wet (Starin, 2002), tree holes and small cavities in tree branches, and tank bromeliads (their phytotelmata, which are commonly filled with water) (Bonvicino, 1989; Martínez et al., 2016).

However, many arboreal species may have limited access to water sources, even in tropical forests, as a result of anthropogenic impacts, such as fragmentation and habitat loss (Kalbitzer and Chapman, 2018). In this context, multiple skills help species to explore different water sources, especially during periods of reduced availability or drought (Albuquerque et al., 2021; Delgado-Martínez et al., 2021). Primates of the genus *Sapajus*, for example, are capable of using tools (e.g., stones and branches) to exploit water resources and ensure their survival (Castro et al., 2017). Considering that the consumption of freely available water is uncommon among arboreal mammals, such as neotropical primates (Campbell et al., 2005), it is important to describe the strategies and skills used by primates to obtain water. In the present study, we report on the water consumption behavior of two neotropical primate species, *Ateles chamek* (Humboldt, 1812) and *Sapajus apella* (Linnaeus, 1758), in the southwestern Brazilian Amazon. Additionally, we contextualized the environmental conditions in which such records were observed and compared them with other records for neotropical primates.

**Figure 1:**
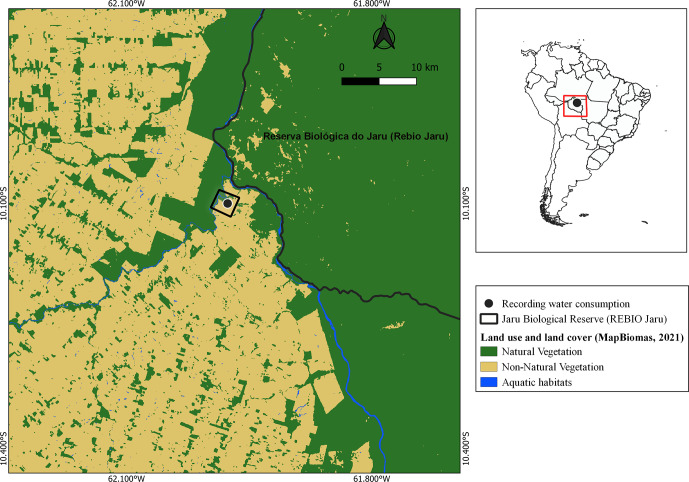
Location of water consumption observations (black point and black square) in the municipality of Vale do Paraíso, Rondônia, the Amazon, Brazil. The square indicates the area in which the two cases of water consumption were observed. The classification of land use and land cover was provided by the open-source MapBiomas project (MapBiomas, 2023).

**Figure 2:**
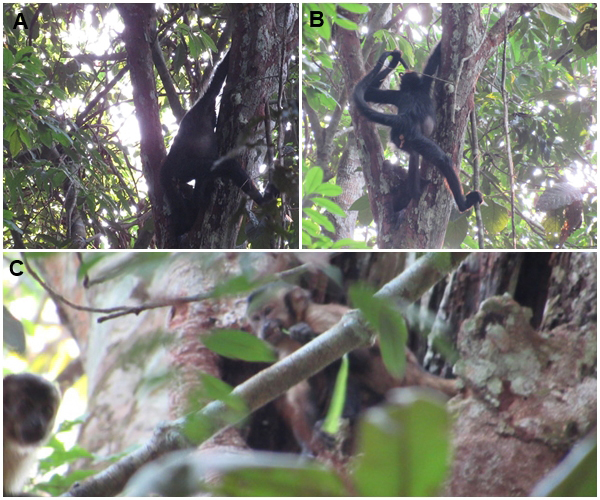
An individual of *Ateles chamek* (**A** and **B**) using its tail to access and consume water from a tree hole, and an individual of *Sapajus apella* (**C**) consuming water from the same tree hole with the aid of a branch with leaves (credit: Luan G. Araujo Goebel).

## 2 Material and methods

Two water consumption events were observed during a species survey in an Amazonian Forest fragment (ca. 52 ha; 10° $05^{\prime }54^{\prime \prime }$ S 61° $58^{\prime }40^{\prime \prime }$ W) in the municipality of Vale do Paraíso, Rondônia, Brazil. The local phytophysiognomy (i.e., vegetation type) is an open ombrophilous forest, characterized by the presence of palm trees, Sororoca, bamboo, and lianas (Perigolo et al., 2017). The forest fragment had an average vegetation height of 10 m (Potapov et al., 2021) and a high density of bamboo (*Guadua* sp. Kunth), surrounded by a matrix composed of pastures bordered by the Jaru River (Veloso et al., 1991). The presence of domestic dogs (Luan G. Araujo Goebel, personal observation, 2022) is an additional threat to the reduced habitat area. The predominant climate is tropical rainy, classified as megathermal (*Aw*, a tropical climate with a dry winter), with a dry season between May and October and a rainy season between November and April (Alvares et al., 2014). The annual precipitation is approximately 1900 mm, and the annual mean temperature is 26 °C.

The forest fragment was monitored for 3 consecutive days in August 2022, between 06:00 and 11:00 AMT and between 13:00 and 18:00 AMT (Amazon time, UTC -4 ), yielding a sampling effort of 30 h. During this period, two primate species (*A. chamek* and *S. apella*) consumed water accumulated in the same hole in a tree trunk, located 16 m high. We used a digital camera to record the species and their respective behaviors. Once recorded, we transcribed the video in the laboratory using All Occurrences (Altmann, 1974) to describe the observed behaviors. Air temperature and relative humidity were recorded hourly using a data logger (HOBO MX2301A; Onset Computer Corporation, Bourne, MA, USA) located at the center of the fragment, approximately 150 m away from the hole. The map was built in QGIS 3.22 LTR software (QGIS Development Team, 2023). The classification of land use and land cover was provided by the open-source MapBiomas project (MapBiomas, 2023), collection 7.1 in raster format (GeoTIFF), with a spatial resolution of 30 m and the WGS84 datum coordinate system.

**Table 1:** Literature records of direct water ingestion events by neotropical primates from arboreal or ground sources.

Primate	Records	Year;	Habitat	Diet	Water	Reference
species		period/season	type		source	
*Sapajus*						
*Sapajus apella*	1	Dry	Ombrophilous forest	Omnivorous	Tree holes	Present study
*Sapajus libidinosus*	1	Dry	Tropical dry forest	Omnivorous	Orchid pseudobulbs;	Castro et al. (2017)
					palm nut; tree holes;	
*Cebus*						
*Cebus capucinus*	2	Dry	Semideciduous forest	Omnivorous	Stream; tree holes	Chapman (1988),
						Gilbert and Stouffer (1989)
*Alouatta*						
*Alouatta belzebul*	1	Rainy	Semideciduous forest	Folivorous	Bromeliads	Bonvicino (1989)
*Alouatta caraya*	2	Hot; rainy	Semideciduous forest	Folivorous;	Tree holes	Bicca-Marques (1992),
				frugivorous		Giudice and Murdry (2000)
*Alouatta guariba*	4	Dry;	Semideciduous forest;	Folivorous;	Stream; bromeliads;	Steinmetz (2001),
		year-round	montane rainforest	frugivorous	hepatic; tree holes	Miranda et al. (2005),
						Moro-Rios et al. (2008),
						Chaves et al. (2021)
*Alouatta palliata*	4	Dry; rainy	Deciduous and	Folivorous;	Stream; ground holes;	Glander (1978), Chapman (1988),
			semideciduous forest	frugivorous	tree holes	Gilbert and Stouffer (1989),
						Serio-Silva and Rico-Gray (2000)
*Alouatta pigra*	1	Hot	Semideciduous forest	Folivorous;	Rainwater; tree holes	Dias et al. (2014)
				insectivorous		
*Ateles*						
*Ateles chamek*	2	Dry	Ombrophilous forest	Folivorous;	Tree holes	Ferrari (1991), present study
				insectivorous		
*Ateles geoffroyi*	4	Dry; rainy;	Ombrophilous and	Folivorous;	Stream; ground holes;	Chapman (1988),
		undefined	semideciduous forest	frugivorous;	tree holes	Gilbert and Stouffer (1989),
				insectivorous		Martínez et al. (2016),
						Delgado-Martínez et al. (2021)

## 3 Results and discussion

On 3 September 2022, three individuals of *Ateles chamek* (two adults and one subadult) were foraging in the same tree. At 16:04 AMT, one adult female suddenly inserted her tail into a hole in the trunk of the tree; when pulling it out, she led the tail to her mouth with the help of her left hand and with the water soaked in her tail – a typical dipping-and-licking behavior (see Wrangham, 1981; Ferrari, 1991) (Fig. 2A–B; see the “Video supplement” section). The female repeated this behavior three times for approximately 2 min, when she then returned to feed on immature leaves of the same tree. After 5 min, the same female returned to the hole, repeating the dipping-and-licking behavior (twice), lasting approximately 2 min. The other individuals in this group continued to feed on immature leaves from the same tree. At the end of the second period of water consumption, all individuals moved perpendicular to the trail to a point where they could not be seen by the observers. At the time, the temperature was 30.67 °C, and the relative humidity was 60 %.

On 4 September 2022, a group of *Sapajus apella* (four adults and two subadults) was scattered while foraging near the same tree mentioned above. At 14:00 AMT, one subadult approached the water source carefully, holding a leaf in his right hand, and inserted his arm into the tree hole. After retracting the arm, the individual consumed the water soaked in the leaf (Fig. 2C). During this time, the individual remained vigilant when looking around. Water consumption lasted approximately 30 s, and he repeated this behavior three times. The individual then joined the group, and they foraged for approximately 10 min. At 14:10 AMT, the group moved out of sight. The temperature was 31.83 °C, and the relative humidity was 58.57 %.

We report on observations of water consumption by two Amazonian primate species. Individuals of both species adopted different strategies to reach the water source, adjusting for their cognitive and behavioral skills. Neotropical primates consume water in different ways, including drinking from arboreal sources, such as tree holes (Ferrari, 1991), as well as terrestrial sources, such as water holes, puddles, and rivers (Chapman, 1988; Bicca-Marques, 1992). To access these sources, primates use strategies ranging from direct consumption to arm-assisted consumption. Hand immersion has been used to access the water by *Alouatta pigra* and *A. caraya* (Giudice and Murdry, 2000; Dias et al., 2014). The use of the tail for water consumption, as observed in the present study, has been previously reported for *A. chamek* (Ferrari, 1991).

However, water consumption with the aid of a tool is uncommon. Primates of the genus *Sapajus* are notoriously capable of using tools (e.g., branches and leaves) for a range of activities (Falótico and Ottoni, 2016), including access to water sources (Castro et al., 2017). In the present study, we observed that *Sapajus apella* accessed the water source with the aid of a leaf and subsequently consumed water from these holes. To the best of our knowledge, this is the first observation of using tools for water drinking for this species. Detailed observations, such as those described here, are essential for understanding the behavior and strategies exhibited by primates to access and consume different sources of water.

The environmental conditions in which these animals consume water are also important. The records reported in the present study were obtained during the dry season and at high temperatures (above the annual mean temperature). Our observations coincided with most existing records of water consumption by neotropical primates, regardless of the type of environment they inhabit (Table 1). During periods of drought, the flowering and fruiting cycles of plants used as food by primates can be affected by a reduction in productivity, quality, and variety, especially in fleshy fruits that contain a greater amount of freely available water (Castro et al., 2017). In addition, during the dry season, there may be changes in the chemical composition of food items, such as increases in dry matter and secondary compounds (Wiederholt and Post, 2010). This implies that a greater volume of water is required for the metabolism of primates. Primate species that are commonly folivorous are more frequently observed for drinking water (Table 1). Such a higher frequency seems to corroborate the idea of a higher metabolic water requirement, as observed by Ferrari (1991) and Jéquier and Constant (2010). The current scenario of increasing fragmentation and mean temperature (see Wiederholt and Post, 2010, and Estrada et al., 2017) may elevate the frequency of freely available water demand by primates. This may represent an additional challenge to the survival of primates and other arboreal species under such conditions.

Choosing to consume water from tree sources can be an important option in fragments that do not have rivers or other water sources. Tree sources can also be a relevant strategy to minimize predation events since descending to the ground can pose a threat and make the individual vulnerable (Mourthé et al., 2007; Ferrari and Hilário, 2012). At the present study site, for example, the presence of domestic dogs represents a further risk, increasing the importance of arboreal water sources. In environments with a low presence of predators, *Ateles geoffroyi* have a higher frequency of ground use for water consumption (Delgado-Martínez et al., 2021). During data sampling, we recorded the presence of humans and domestic dogs moving through the forest fragment. This represents a risk to primates, limiting the descent of these animals to the ground to consume water directly from the existing river at the study site. Going down to the ground in anthropized areas, such as this, is a challenge to survival, being only observed in cases of extreme necessity, as reported by Mourthé et al. (2007). Thus, arboreal water sources are of great importance to the survival of arboreal species, especially in the dry season (Dias et al., 2014) and when there is a risk of predation due to anthropogenic factors.

## 4 Conclusions

Our observations reveal the behaviors and tools used in water consumption by two primate species. Reporting such behaviors, as in the present study, is relevant to the ecological knowledge of the target species, helping to understand how primates adjust their behavior to obtain food and nutritional resources. More investigations are needed to associate the budget of primate activities and water consumption with environmental characteristics, such as fragmentation conditions, seasonality, and the presence and abundance of potential predators. In addition, further studies should investigate the consequences of anthropogenic impacts and climate change on primate species, thus improving conservation strategies for primate species and other arboreal mammal species.

## Data Availability

The water consumption data for *Ateles chamek* and *Sapajus apella* described in this article can be found in Table 1.

## Video supplement

The video footage of water consumption is available under the following 10.5446/68527 (Goebel et al., 2024).
